# Traumatic brain injury and immunological outcomes: the double-edged killer

**DOI:** 10.2144/fsoa-2023-0037

**Published:** 2023-05-09

**Authors:** Souvik Datta, Feng Lin, Lawrence D Jones, Sandeep C Pingle, Santosh Kesari, Shashaanka Ashili

**Affiliations:** 1 Rhenix Lifesciences, 237 Arsha Apartments, Kalyan Nagar, Hyderabad, TG 500038, India; 2 CureScience, 5820 Oberlin Drive #202, San Diego, CA 92121, USA; 3 Saint John's Cancer Institute, Santa Monica, CA 90404, USA

**Keywords:** immune response, immunopathology, management protocols, neuroinflammation, risk factors, traumatic brain injury (TBI)

## Abstract

Traumatic brain injury (TBI) is a significant cause of mortality and morbidity worldwide resulting from falls, car accidents, sports, and blast injuries. TBI is characterized by severe, life-threatening consequences due to neuroinflammation in the brain. Contact and collision sports lead to higher disability and death rates among young adults. Unfortunately, no therapy or drug protocol currently addresses the complex pathophysiology of TBI, leading to the long-term chronic neuroinflammatory assaults. However, the immune response plays a crucial role in tissue-level injury repair. This review aims to provide a better understanding of TBI's immunobiology and management protocols from an immunopathological perspective. It further elaborates on the risk factors, disease outcomes, and preclinical studies to design precisely targeted interventions for enhancing TBI outcomes.

## Plain language summary

Traumatic brain injury (TBI) is a leading cause of death and disability worldwide due to falls, car accidents, sports and blast injuries. TBI causes severe, life-threatening consequences due to inflammation in the brain. Unfortunately, no current therapy or drug protocol can address the complexity of TBI, leading to long-term chronic inflammation. However, the immune response plays a crucial role in repairing injured brain tissue. This review aims to provide a better understanding of TBI's immunobiology and management protocols to design targeted interventions for better outcomes in TBI patients.

## Tweetable abstract

Traumatic brain injury (TBI) causes severe consequences, but no current therapies target its complex pathophysiology. Our review examines the immune response's role in tissue repair and proposes immunopathological strategies for TBI management.

Traumatic brain injury (TBI) is notably an insult to the brain from an external force. The result of such force may be temporary or permanent impairment of normal brain function. TBI can be classified based on type (non-penetrating or penetrating head injury) and severity (from mild to severe) [1,2]. TBI is one of the leading causes of death and disability worldwide. The injury is primarily associated with neuroinflammation due to immunological reactions. Secondary systemic inflammations are having long-lasting effects after a primary brain injury depending on the site and extent of primary damage [3]. The low-income and middle-income countries specifically show higher rates of morbidity as well as mortality than high-income countries [4]. However, the strict enforcement of seatbelts legislation, helmet use, and safety regulations as well as early intervention in the form of neurointensive care during emergencies contribute to reduced TBI fatalities in first-world countries [4]. According to the US Centers for Disease Control and Prevention [5], for much of 2009–2018, mortality rates of TBI were highest for persons belonging to the age group of 20–24 years [5]. The highest rates of traumatic brain injury (all forms) dependent hospitalization and death are seen in vehicular-based accidents. Approximately 2.5–6.5 million US citizens are living with TBI-related disabilities at a very high healthcare cost and a severe economic burden due to longlasting and expensive management protocols to reduce the immunological sequel of the ailment [6].

There are innumerable modes of acquired injury including extrinsic compression from a mass lesion, diffuse axonal injury, contusion due to accidents, and some intrinsic mechanisms by which neuronal injury can be caused such as ‘classical’ ischemia, apoptosis, mitochondrial dysfunction, cortical spreading depression or CSD, and microvascular thrombosis [4,5]. The clinical stratification of traumatic brain injury has been done based on post-resuscitation Glasgow Coma Scale (GCS) scores. According to GCS scores (3–15), there are three stratifications as severe (GCS 3–8), moderate (GCS 9–13), and mild (GCS 14–15) TBI, respectively [6–10]. Trauma centers have played a crucial role in reducing the rate of mortality when treating severe cases of TBI (GCS <8) [11]. Furthermore, TBI mortality is higher in countries where resources are limited, particularly among young males [12]. One cohort-based study suggested using the MYSTIC-score (mortality in moderate–severe TBI plus ICU-complications) as the first-hand tool to predict the risk of mortality in the hospital among moderate to severe TBI patients with ICU complications [13]. By 2019, figures suggested that in the USA alone, there were approximately 1.7 million annual cases [14]. Further, the bidirectional sequel of TBI immunology involves both the repairment of tissue injury and immunological inflammations to result in various degrees of neurophysiological outcomes. The current review explains the important areas of such double-edged TBI outcomes, factors as well as management issues for TBI. Further, the review will focus on the immunological cascades and various disease outcomes which can be targeted in designing successful phase III–IV clinical trials in cohort-based studies to design robust therapeutics.

In this study's literature review, we utilized the MeSH term ‘traumatic brain injury immunology outcomes’ to conduct a comprehensive search. This research yielded over 300 pieces of literature. We also used additional MeSH terms like ‘primary traumatic brain injury’ and ‘secondary traumatic brain injury’, which resulted in over 6000 publications in the MEDLINE database via PubMed. However, we filtered the results for updated literature and found only 600 relevant pieces published within the last year. This highlights the need to better understand the range of outcomes to design more precise therapeutics, which is the primary objective of this review. We finalized our search with all relevant literature from the database and supplemented it searching clinical trial registration databases (clinicaltrials.gov) for relevant trials from inception to January 2022. Overall, our search yielded results including reviews, clinical trials, meta-analyses and systemic reviews.

## Various outcomes associated with a primary injury

The neuroimmunological damage does not occur immediately after primary brain injury. Most of the immunological damage from TBI occurs days and years following injury [15]. The irreversible nature of primary mechanical injury results in cerebral damage or neurotrauma with cerebral edema, which contributes to secondary injury from raised intracranial pressure (ICP), decreased blood pressure, anemia, hypoxia, seizures, hypoglycemia as well as hyperthermia. The post-injury phase of a severe cerebral blow can result in immediate cell death of neurons and glial cells (mainly necrosis) along with axonal injury, and disruption of the blood–brain barrier (BBB). Further edema and the release of damage associated molecular patterns or DAMPs and excitotoxic agents are critical factors for initiating systemic inflammations [16]. Immediate management of such complications needs to be addressed with prompt actions [12].

Primary injury can result in focal and diffused lesions. The GCS score reflects the risk of dying from primary TBI. The risk of death is low after an individual gets mild TBI (∼1%), whereas the risk of death increases up to 15% for moderate TBI and up to as high as 40% for death after severe TBI [15]. The focal lesions are mainly neuronal lesions in the very focus of impact from external contact sources. They are epidural hematomas, cerebral contusions, and subdural hematomas. An epidural hematoma is a result of head trauma linked to lateral skull fracture as well as tearing of the middle meningeal blood vessels [14]. Cerebral contusion is a kind of tissue bruise and hemorrhage due to leakage of the blood vessels in the brain. Subdural hematoma is characteristic of the build-up of blood on the surface of the brain. Diffused lesions area including diffuse axonal injury, is often seen in cases of injury caused by rapid acceleration-deceleration or rotational external force. TBI patients with diffuse axonal injury usually have high rates of both morbidity and mortality [17]. Traumatic subarachnoid hemorrhage can be an outcome of both focal damage as well as diffuse vascular injury [15].

Both focal and diffuse brain injury can lead to coma and possible vegetative state. As the intensity and impact of mechanical force triggering TBI vary, the outcomes also become heterogeneous based on the extent and site of damage. Severe TBI may go through a an unconscious and consequent vegetative state that manifests in the form of a paralytic day-night cycle, with the opening of the eye, command disobediences as well as lack of speech production. The subsequent phase of the recovery process can vary from minutes to months and is exhibited by a period of disorientation, memory disorders, and behavioral disturbances (post-traumatic amnesia or PTA). Consequently, the succeeding phase of recovery for a TBI patient is characterized by a repertoire of cognitive, mood, behavioral, and sensorimotor anomalies, which are the main neuroimmunological cascades involving the frontal and temporal lobes of the brain being the most affected brain regions [15].

Cortical contusions of the frontal-temporal brain regions of the brain due to the sudden movement against the skull base lead to frequent impairments. Such impairments include lack of attention, concentration, memory, and deficits post-TBI [18]. In addition to the focal injury, following diffuse axonal injury, impairments in memory and executive functioning are also common [19,20], whereas, the incidence of persisting motor weakness post-TBI is lower [15,21]. A concussive injury is an immediate non-structurally damaging injury that is characterized by fluctuating levels of transient altered mental status with the mental status manifesting from slight confusion to an actual state of unconsciousness, but with recovery occurring in minutes [22].

## TBI assessment & progression to secondary damage

The secondary damage follows primary outcomes after days to even years. Neurogenic inflammation is the pivotal immunological cascade following TBI [23]. The primary outcomes trigger brain herniation syndromes, edema, cranial nerve deficits, infarction, and cerebral ischemia [24]. In general, secondary injury or damage refers to the cascade of cellular and molecular processes due to the primary injury. It also consists of cerebral damage due to hypoglycemia, hypotensive or hypoxic events, and raised intracranial pressure or ICP [15]. The TBI survivors incurring moderate to severe are at increased risk for cognitive, behavioral as well as emotional problems in the form of secondary damage. Clinical evidence further suggested that significantly more emotional dysregulation, and attentional, and psychological difficulties are found in TBI individuals in comparison to controls [25]. The report showed despite the resolution of the acute complications following TBI, there is substantial neuropsychiatric and cognitive impairment as well [26].

Molecular and cellular expansions of primary damage as indirect damage are critical to appreciating secondary injury in TBI. However, the immune response to TBI is intended by the body's projected strategy to attain neuroprotection and further repair caused by the damage, but it can often go out of proportion leading to the severity of inflammatory processes. The important immunological outcome that leads to either repair of function or further collapse of function, depends on the nature, duration, and magnitude of the primary immunological events that develop post-brain injury. Following dysregulation, the immune system can provoke a secondary phase of chronic tissue damage as well as neuroinflammation [27]. The secondary sequel at the cellular level is cellular hyperexcitability, vasogenic as well as cytotoxic edema, hypoxia-ischemia, oxidative stress, neuroinflammation, cell death, and tissue degeneration [28]. Astrogliosis is a common event related to neuronal death in brain injury. Inflammatory cytokines, neurotoxic molecules, and chemokines play a major role in neuronal damage [29]. Astrocytes are associated cells in the brain that act as primary responders to CNS assaults via the multitude of potential changes that are popularly referred to as reactive astrogliosis [30].

TBI is characterized by apoptotic and necrotic cell death of neurons and glia respectively, along with disruption of the BBB with an additional infiltration of the injured brain by peripherally-derived immune cell populations such as neutrophils and macrophages [31]. There is an enhanced acceptance that TBI not only has immediate clinical consequences but also has the potential for gradually evolving long-term sequel of immunological outcomes leading to increased susceptibility for the development of future neuropsychiatric disturbances along with seizure disorders and/or neurodegenerative diseases such as Parkinson's or Alzheimer's disease [32–35]. Single-cell RNA sequencing depicted the nature of microglial cell depletion, neuropathogenesis, and long-term functional impairment of neurons in mice models of the sub-acute and chronic TBI [36]. Moreover, TBI-induced inflammations have been strongly associated with the development of other neurological disorders involving anxiety, depression, post-traumatic stress disorder (PTSD), chronic traumatic encephalopathy (CTE), and amyotrophic lateral sclerosis [3,37–39]. CTE has especially drawn national attention due to its distressing effects on professional players of collision sports such as American football [40]. The cognitive decline studied in the mice model is exaggerated microglia-mediated inflammation along with amplified IL-1β, and TNF-α, CCL2 chemokine as well as prolonged TNF-α cytokine/chemokine expression, and a distinct reactive morphological profile of microglia [26]. Another secondary effect after traumatic brain injury is gastrointestinal problems as observed in the mice model. A review of the literature demonstrates the communication of the CNS to the gut linked to post-injury dysbiosis, gastrointestinal-associated lymphoid tissue-mediated neuroinflammatory responses, as well as bacterial-metabolite neurotransmission [41].

## Risk Factors affecting elevated immunological sequel after traumatic injury

There are certain risk factors associated with the development of long-term secondary effects in TBI patients. TBI significantly enhances the risk of suicide and is the major cause of acquired seizure disorders [40]. Age, education, traumatic axonal injury (TAI), and depression appear to elevate the risk of poor quality of life for TBI patients, indicating the need for long-term follow-up of such patients [25]. Age predisposes intensified the inflammatory response post-TBI when there was enhanced recruitment and activation of peripheral monocytes following infiltration to the injured brain site leading to neuronal damage and major impairment [42,43]. When considering the posture-related development of a shear strain of the head due to primary impact, an essential factor is the direction of strain development. The development of the strain due to lateral head movement is linked with more severe diffuse damage than sagittal head movement [44]. A literature review suggests that head contact of mechanical impact has a significant additive effect on the development of shear strain levels and further outcomes [15,45,46].

The molecular factors for induction of secondary TBI following primary injury are critical to addressing better management approaches in the long term. The responsible molecular factors experimented on model animals showed contribution to the phenomenon which consists of metabolic changes, edema formation, calcium influx, increased oxidative stress, excitotoxicity, mitochondrial dysfunction, lipid peroxidation, neuroinflammation, and ultimately, cell death via necrosis or apoptosis at the molecular level [47,48]. Inflammatory responses determine much of the secondary brain cell injury such as induced oxidative stress as well as edema formation [49–51]. Males are at a greater risk for elevated immune response after TBI as seen in the mice model through macrophage/microglia activation [52].

## Immunopathogenesis

The chronic inflammatory processes are varied which can subsequently lead to secondary death of neuronal cells, neurodegeneration, as well as persisting neurological impairment [27]. Although some extent of inflammation is needed for fine-tuning the requisite for tissue repair by the body's immune system but exaggerated and misdirected immune system brings assault leading to neuroinflammation and degeneration. Cytokines storm is a distinct feature of immunology in TBI. One of the major functions of the body's immune system is to restore homeostasis through repair mechanisms following injury and tissue damage. Critical functions of the immune system responding to external injury in TBI include phagocytosis (disposal) of cell debris following sequestration of tissue-damaging molecules, as well as the enhancement of the wound-healing response for neuronal repair and restoration [27]. Interestingly, astrocytes accompany neurons and glial cells in the brain having beneficial and detrimental effects on TBI pathophysiology. These include the promotion and restriction of neurogenesis and synaptogenesis, both acceleration and suppression of neuroinflammation, as well as regulation of the BBB via multiple bioactive factors. Astrocytic-derived factors such as aquaporin-4 are contributory to the formation of cytotoxic edema in TBI patients [53]. The resident cells after getting the alarm signals from immediate damage signals direct neutrophils and macrophages through enhanced phagocytosis and necrosis in the site of injury following the mechanical stimuli in TBI. Further, the cellular infiltrations in the injury site are composed of microglial cells that are continuously under active surveillance of the CNS (central nervous system) compartment to identify structural anomalies. When neutrophil numbers begin to decline, the activated microglia usually assemble at the injury to facilitate restorative processes. Further, the restorative and repair process involves monocytes coming to the injury site, and releasing chemokines to recruit astrocytes [54].

Immunology and inflammation involve the arm of innate immunity and further adaptive immunity. Inflammation involves inflammasome-activation and release of cytokines (TNF-α, IFN- γ, IL-1β as well as IL-10), chemokines (CCL2, CCL5, CXCL9), and inflammatory mediators such as Type-I Interferon, complement proteins, GM-CSF, and nitric oxide. The inflammatory factors further direct the infiltration, recruitment, clonal expansion, and subsequent survival of peripheral immune cells such as the dendritic cells, macrophages, T cells, and B cells within the site of injured brain tissue [31]. Primarily, the various external traumatic stimuli trigger the activation of resident microglial cells followed by the infiltration of peripheral macrophages in TBI. These are the foremost neuroinflammatory responses that are directly having the outcome of neuronal death due to reduced oxygen supply leading to mitochondrial dysfunction and intracellular accumulation of lactic acid [52,55,56].

Primary tissue damage that results from cerebral trauma elicits damage-associated molecular patterns (DAMPs) such as ATP, reactive oxygen species (ROS), damaged mitochondria, and necrotic cells as well as alarmins like interleukin (IL-1α, IL-33, and HMGB1). Various immune receptors recognize the DAMPs to stimulate the localized release of cytokines and chemokines at the site of injury. Subsequently, the localized events follow coordinated activation, expansion, and recruitment of infiltrating lymphocytes to the site of damage [27]. The BBB permeability changes drastically following TBI involving increased transport of blood plasma proteins like albumin (transcellular) through the activation of caveolae, which further activates microglia and astrocytes. The shear stress in primary brain injury evokes the release of special neuropeptides like substance P (SP) by transient receptor potential cation channels or TRP channels (e.g., TRPV1 and TRPA1). The SP further triggers many facets of the inflammatory responses through the activation of microglia and astrocytes, promoting white blood cell migration and degranulation of mast cells. SP also set off the earliest changes observed in BBB before the decline in expression of tight-junction claudin-5 and occluding proteins [23]. Triggering receptors expressed on myeloid cells 2 (TREM2) also has implications for the development of neurodegenerations following TBI [57].

The cellular oxidative stress in the form of the generation of ROS and reactive nitrogen species (RNS) directly activates the release of inflammatory cytokines IL-1β, TNF-α and TGF-β. The ROS and RNS indirectly activate the metalloprotease enzymes such as MMPs [58]. The oxidative stress further leads to the degradation of VEGFR-2 through MMPs. This results in a decline in VEGFR-2 with a subsequent increase in VEGF-A level leading to cellular apoptosis and neuroinflammation through the activation of caspase-1/3 and IL-1β release [58]. In TBI failing to repair and restore the CNS homeostasis, there is persistent chronic microglial activation and further production of pro-inflammatory mediators such as ROS, complement protein C1q, IFN-γ, and IL-1β resulting in successive neuronal cell death [31]. Long non-coding RNAs are also involved intracellularly for bringing neuroinflammatory changes as observed in animal models with *HOXA11-AS* RNA, which can aggravate neuroinflammation after TBI [59].

There is a major link between the progressions of TBI and to development of Alzheimer's disease [60] as a secondary outcome. It is suggested that amyloid β plaques (Aβ) responsible for the pathology of the development of AD, are initiated by traumatic brain injury [61]. Further, both tau protein accumulation which is another causal pathological agent in AD is also emphasizing the role post-traumatic injury [62]. A breakthrough occurs in the understanding of a critical issue of reduced immunology associated with TBI. Recently, it is speculated that TBI may also change the systemic immune response of an individual to make that person more vulnerable to infections during the acute post-injury period. The flip side of immunosuppression following TBI may result in opportunistic infections posing an additional challenge to the patient. In addition, hospitalization, surgical interventions, and such a state of immunosuppression to the CNS, may all add to the high rate of infections such as pneumonia in TBI patients [63]. There is increasing evidence on the role of CCR-2, a receptor of chemokine involved in regulating the infiltration of monocytes in inflammations and degenerations after TBI. However, studies on the mice model suggested the dichotomous role of the chemokine receptor in regulating monocyte infiltration, tau protein phosphorylation, and further axonal damage [64]. The other arm of immunity called the adaptive immune response is subsequently activated in TBI patients leading to the common and chronic effects of inflammation of the neuronal system [65].

## Current management

TBI has complex and heterogeneous manifestations of immunological consequences demanding immediate and precise management. Immediate hospitalization and intensive care are required for monitoring the vital parameters after the initial injury due to neurotraumatic episodes. Despite the multitude of pre-clinical and clinical research, treatment options to date for TBI depends heavily on supportive care with very limited targeted interventions to manage both acute and chronic immunological sequelae of TBI [65]. Therapies for TBI can have a continuum starting from medical management alone with frequent neurological exams, to invasive monitoring of intracranial pressure, and ultimately to radical decompressive surgery [22].

As the primary injury is irreversible, management strategies must be emphasized on preventing secondary immunological consequences by avoiding physiological states such as dropped blood pressure and hypoxia and maintaining cerebral perfusion pressure (CPP) [66]. In an emergency scenario, CPP can be sustained by elevating the mean arterial pressure (MAP) or decreasing intracranial pressure (ICP), or the use of both parameters regulation. A combination of pressors is employed to enhance the MAP for patients in the euvolemic state. Further, an algorithmic approach utilization based on simple bedside maneuvers practice, hyperosmolar therapy, and cerebral spinal fluid (CSF) drainage can be used for controlling life-threatening elevated ICP in TBI patients. Pentobarbital infused coma and decompressive craniectomy can be advised in refractory cases of TBI. If mass lesions occur post-injury in the brain, it requires operative evacuation based on exam findings, as well as ICP measurements [66]. In addition to these important considerations, intensive care such as mechanical ventilation, appropriate blood product transfusion, hemodynamic stabilization, management of paroxysmal sympathetic hyperactivity (using nutrition as a therapy), venous thromboembolism as well as seizure prevention is all essential in moderate to severe TBI patient management, which is described in the 2016 Brain Trauma Foundation Guidelines [6]. Clinicians put focus on eye-opening as one individual component of the GCS grading that is suggestive of a more reassuring neurological status if a patient opens his or her eyes spontaneously. For the breathing difficulties of intubated and drug/alcohol-intoxicated patients, usually, sedatives and pharmacologically induced paralysis are performed for placement of the endotracheal tube [22]. The infants and children with TBI, therapeutic hypothermia has been shown to decrease oxidative injury as temperature brings down the cerebral metabolism demand decline [67]. However, therapeutic hypothermia also originates with risks like unwanted alterations in blood sugar, platelet count, as well as coagulation factors. Thus, the platelet count and coagulation factors must especially be checked before an individual is brought to a hypothermic state. Cerebral perfusion pressure and brain oxygenation are other important metrics during the acute hospitalization phase of TBI patients. Electroencephalogram (EEG) may also be required to assess sleep/wake cycles to evaluate seizures [1].

Neuropsychiatric complications are very common and require professional help after TBI. In critical care settings, there are FDA-approved medications for the treatment of acute neuropsychiatric symptoms post-TBI. The harmful effects of psycho-pharmacologic agents, like benzodiazepines and antipsychotics, used in the treatment of TBI patients, especially on children must be considered. In case of cognitive deficits after a TBI, dopaminergic agents and psychostimulants may be administered. On the other hand, patients suffering from delirium post-TBI are usually treated with alpha-2 antagonistic agents, anticonvulsants and antipsychotics [68].

For bone fracture cases especially in children, if the autologous bone flap cannot be utilized, alternative titanium implant materials may be used [69,70]. The involvement of the neurosurgical and rehabilitative team should be of utmost importance for rapid action regarding bone flap replacement. Even before bone flap replacement, the protective headgear may be recommended based on the preference of the physician as well as patient safety needs. Before cranioplasty, the site of the absent bone flap should be monitored for fluid levels, which may indicate complications such as clots, seizures and hemodynamic instabilities as well as paralysis. Thus, the absent bone flap and the replacement therapy of the bone segment is a critical process for families, along with surgeons [71].

There are calcium channel blockers, corticosteroids, mannitol, magnesium, excitatory amino acid inhibitors, progesterone, monoaminergic agonists, recombinant factor VIIa, complement system inhibitors, and free radical scavengers that are commonly used as drug-based therapeutic interventions in TBI treatment for decades. The cell-based therapeutic products include monoclonal antibodies, erythropoietin and its carbamylated form, statins, bone marrow stromal cells, stem cells, or in combination or with biomaterials which are used to attain neuroprotection and promote brain remodeling [72]. According to the first Guidelines for Management of TBI (1995) and its adherence, the therapeutic management of TBI has achieved substantial improvement in functional outcome scores and reduced mortality rate, length of hospital stay, as well as the cost of bearing the sustenance and quality of life [73]. The inclusion of cell-based therapeutics and cellular products such as erythropoietin, carbamylated form of EPO (CEPO), and bone marrow stromal cells contribute to a major development in the treatment avenues of TBI. In addition, the utilization of methylphenidate, progesterone, statins, dexanabinol and rivastigmine has gained popularity in TBI management [72]. To date, experimental investigations and clinical trials have especially emphasized the neuroprotective and restorative strategies of TBI therapeutics with the purpose to reduce secondary brain damage [72]. Several immunological pathways are targeted for designing novel pre-clinical therapeutics in TBI as targeted therapy [74] Specifically, glutamatergic and GABAnergic pathways are targeted with emphasis on NMDA receptors, neuronal synapse receptors and autophagy receptors as targets. Further, emerging therapeutic aspects involves targeted immunotherapy with anti-HMGBt monoclonal antibodies, barbiturates, and glibenclamides to reduce brain edema. Some more targeted inhibitors like Kollidon VA64, citicoline as calpain inhibitor, FK506 (calcinurin inhibitor) and VAS203 (NO synthase inhibitor) have been promising in reducing traumatic axonal injury. In addition, the development of cellular therapies has further shown precise modalities in the management of TBI (Table 1).

**Table 1.** Some promising targeted therapeutics, and approaches in TBI.

Pathways	Major targets	Promising therapeutics	Preclinical Effect	Ref.
Glutamatergic Pathways, neuroptosis, autophagy, lysosomal pathway, excitotoxicity pathway, GABAnergic pathway	NMDA receptor, neuronal synapse receptor, autophagy receptor, caspase enzyme	MK801, Ro25 6981, Memantine, 3-methyladenosine, Levetirecetam, Caspase-1 inhibitor	Prevent excitotoxicity, and neuronal death	[75–81]
RAGE pathway, BBB permeability, cytokine releasing pathway	HMGB-1, IL-6, intracranial pressure, sulphonylurea receptor	Anti-HMGB1 monoclonal antibody, ethylpyruvate, mannitol, glycyrohizin, barbiturates, glibenclamide	Reduce brain edema	[82–91]
Redox regulatory pathway, oxidative phosphorylation, electron transport chain, apoptotic pathway	MPTP, TIR-4, HMGB1, HMG-CoA reductase enzyme	Antioxidant as PEG-SOD enzyme, U83836E, edaravone, MPTP inhibitors, SS-31 peptides, Mito-Q, GS-nitroxide as redox regulators, rosuvastatin, dimethyl fumerate, glatiramer acetate as scavenger of cytotoxic molecules, low hanging fruit, neutraceuticals	Mitochondrial regulation, oxidative stress reduction, neuroprotection	[92–97]
Cytoskeleton stabilization, cytochrome P450 pathway, mitochondrial calcium channel pathway, Rho GTPase and kinase pathway	Chemical depolymerisation, membrane disruption, calpain, calcinurin, NO synthase, arachidonic acid metabolite formation, calcium overload	Depolymerization inhibitor Kollidon VA64, citicoline as calpain inhibitor, FK506, VAS203, inhaled NO as vasodilator, ET-1 antagonist, CsA, DNA Vaccine against myelin-derived axonal growth inhibitors	Traumatic axonal injury reduction	[98–102]
Dopaminergic pathway, neuroinflammatory pathway promoting astrogliosis	D2 receptor, dopamine transporter, neuronal transport	Perfluorocarbon-enhanced oxygen delivery, methylphenidate, dopaminergic drug amantidine, D2 receptor agonist as bromocriptine	Reduce inflammation, cerebral ischemia, cognitive enhancement	[103–105]
Cellular neuroprotective pathways, augmentation of endogenous pathways	VEGF, BDNF, NGF, bFGF, EGF	Stress regulation by RBM3 inhibition, exogenous bone marrow derived stem cells enhancing neurogenesis, bone marrow derived stromal cells, intracranial human bone marrow stromal cells in collagen scaffold, adipose-derived stem cells, neuroprotective agent P730-A20	Cellular therapies, neurotropic factor enhancement therapies	[106–109]
Cerebral blood flow, tissue perfusion, routed drug delivery	Augmented agent for blood flow	PNPH resuscitation solution, drag reducing polymers, colloid small molecules, albumin, vesicular delivery, small penetrating peptide based delivery, nanocarriers and osmotic pump based delivery	TBI resuscitation, tissue perfusions, ICP reduction, edema reduction, and better targeted drug delivery	[47,110–115]

BBB: Blood–brain barrier; ICP: Intracranial pressure; TBI: Traumatic brain injury.

In the field of managing patients post-TBI, rehabilitation plays a huge role. Rehabilitation of TBI patients is concentrated primarily on using alternative strategies to manage comorbidities and minimize complications after injury. For children, the involvement of caregivers and parents to achieve reintegration into the home, school and community life is a common goal. Reintegration gains momentum when the child's previous school system initiates building contact with educators to gather background information about the child's previous level of functioning. This information is valued very much to provide the right support to the child and family members. After admission to inpatient rehabilitation, evaluation of the child's functional independence is the preferred criterion for TBI sufferers using the WeeFIM II System (Uniform Data System of Medical Rehabilitation, Amherst, NY). The functional quality and independence are measured by an 18-item performance-based instrument in comparison to peers having similar ages for ages 6 months to 7 years of age [71]. In addition to the immediate primary care, there is a further need to establish a constant medical home and ongoing outpatient care to sustain the quality of life for the child in a local area near the home.

## Preventive measures

Awareness of preventive measures continues to take a lead role in reducing the number of TBI victims. For example, instituting protective gear in the form of lateral side airbags and helmets as preventive measures for vehicles to avoid head injury and accidents [15,73]. These public health initiatives of encouraging the use of seatbelts and airbags as they have a direct impact on reducing mortalities associated with TBI [116]. However, after primary injury, it is also crucial to prevent secondary immunological assaults post-TBI. Increasing awareness has been the focus of the CDC. Through the initiatives of the CDC, an effort to assess TBI primary and secondary immunological outcomes, creating enhanced brain injury awareness, and tackling ways to prevent TBI through motor vehicle safety rules are major areas to consider [117]. In addition, the increased awareness and use of sports helmets in collision sports were seen to avoid severe injuries to the brain [118]. Growing awareness and education regarding concussions in sports may help detect TBIs earlier, and aid in early management to prevent deteriorating on a long-term basis and life-threatening conditions [74,116].

## Conclusion

Immediate access to trauma centers and emergency centers is critical to reducing the future medical outcomes of TBI patients. Prompt response and management are required for preventing secondary injury. Although there is currently a lack of targeted therapeutics and a sustainable treatment program for TBI recovery to date, there have been continuous efforts for developing novel therapeutic strategies for TBI. However, the improved understanding of the repertoire of clinical manifestations as well as complicated pathophysiological mechanisms of TBI has paved way for the development of novel targeted therapeutic interventions through preclinical studies and phase I/II trials. But the majority of the trials turned out to be a failure in phase III clinical trials in the last few decades. Thus new directions of preclinical studies on targeted therapeutics and precision approaches of marker diagnosis as well as prognosis call for TBI research.

## Future directions & perspective

Astrocytes are involved in bringing both functions of tissue repairment-restoration as well as neurodegeneration, targeting therapeutics on the pathways controlling astrocytes showing plasticity and reconstruction can be promising for TBI victims [119]. As the primary impact of immunological cascades on the site of injury involves the release of widespread cytokines and chemokines for cellular communication, this can be another useful target for therapeutic interventions. TBI therapeutics also depend on the extent and site of brain injury. Modulating chemokine signaling can be a future approach to ameliorating TBI [120]. Phenotypic screening and precision approaches are coming into future directions as a mode of personalized medicine approach in the treatment of TBI [121]. Some of the important and emerging ongoing clinical trials are enlisted in Table 2.

**Table 2.** Ongoing clinical trials in TBI.

Conditions	Therapeutics/therapy	Trial phase	Clinical registry ID
Mild TBI	Hyperbaric oxygen therapy	III	NCT02089594
Lower limb or upper limb spasticity associated to TBI	NT 201 (botulinum toxin)	III	NCT03992404
Minor TBI	S100B protein	III	NCT03780062
TBI	Umbillical cord-derived MSC	I	NCT05018832
TBI	PNT001	I	NCT04677829
TBI	Autologous MSC	I, II	NCT04063215
Mild TBI	Exercise as concussion therapy	II	NCT04578743
TBI	Hyperbaric oxygen treatment	II	NCT02407028
Adult severe TBI	Antisecretory factor given as a food supplement	III	NCT03339505
Post TBI vasogenic pericontusional edema	Dexamethasone	III	NCT04303065
Severe TBI	Antisecretory factor (Salovum)	II	NCT04117672
TBI	Amantadine	IV	NCT04527289
TBI associated fatigue and cognitive impairment	Growth hormone therapy (Somatropin)	III	NCT03554265
TBI	Human C1 inhibitor (@Fter)	II	NCT04489160
Epileptic trauma in TBI	VPA	I	NCT02027987
Mild TBI	NeuroAiD II™ (MLC901)	III	NCT04861688
TBI-PTSD comorbidity	Cannabidiol	II	NCT04550377
TBI	Autologous stem cell therapy	II	NCT02525432
Concussion associated to TBI	Ghrelin (OXE-103)	II	NCT04558346
Mild adult TBI	Tranexamic acid	III	NCT04521881
Pediatric TBI	Nucleo CMP forte	III	NCT04499755
TBI	Dexmedetomidine	IV	NCT04006054
Pain, seizures, stress associated to chronic TBI	Cannabis	II	NCT03944447
Memory deficits associated to TBI	Donepezil	III	NCT02255799
TBI related pain	Ketamine adjunct therapy	IV	NCT05097261
Post pediatric TBI concussion syndrome, headache	Nortriptyline	IV	NCT04226365
TBI	Anodal transcranial direct current stimulation	II	NCT02849223
Un- consciousness related to TBI	Methylphenidate	I	NCT03814356
Adult upper limb spasticity related to TBI	IPN10200	I, II	NCT04752774
Alcohol use disorder and stress related to TBI	repetitive transcranial magnetic stimulation	II	NCT03995173
TBI	Aerobic exercise and rehabilitation	I	NCT03407924
TBI related headache	Prazosin hydrochloride	I, II	NCT02266329
Functional disability related to mild TBI	Magventure MagProX100 with MagOption stimulator and Magpro Cool Coil B65 A/P device	II	NCT04043442
Concussion related TBI in retired football players	Growth hormone therapy	II	NCT04121780
TBI	Biperiden Lactate	III	NCT01048138
Moderate to severe TBI	MLC901	IV	NCT04487275
Spasticity related to TBI	Rimabotulinum toxin B	II, III	NCT04099667
Moderate TBI	Dietary supplement: MLC901	IV	NCT04766281
TBI	Vegal nerve stimulation	IV	NCT04437498
TBI	Nitric oxide	III	NCT03260569
Post-concussion syndrome related to TBI	Adipose-derived stem cell infusion	I	NCT04744051
Mild to severe TBI	Beta blocker propranolol	IV	NCT04508244
Non-penetrating moderately severe TBI	Cytoflavin (inosine + nicotinamide + riboflavin + succinic acid)	III	NCT04631484
Thromboembolic issues related to TBI	Dalteparin	III	NCT03559114
Critically ill penetrating TBI	Epoetin alfa	III	NCT04588311

TBI: Traumatic brain injury.

The future years will dedicatedly see the prospects of targeted immunotherapy and personalized approach in dealing with each TBI patients. Moreover, in the coming ten years, biomarker based non-invasive diagnostics and cellular therapies needs to be implemented to take care in rapid management of patients.

**Executive summary**

- Traumatic brain injury (TBI) causes death and disability globally via neuroinflammation.
- Glasgow Coma Scale (GCS) stratification and trauma centers reduce TBI mortality.
- TBI immunology involves both tissue repair and inflammation with varying neurophysiological outcomes.

**Various outcomes associated with a primary injury**

- Primary brain injury can cause cerebral edema, intracranial pressure, and neuronal/glial cell damage. Urgent management is essential.
- TBI severity can lead to focal and diffused lesions, coma, cognitive and behavioral impairments, cortical contusions, and executive and memory dysfunction.

**TBI assessment**

- TBI can cause long-term cognitive, behavioral, and emotional problems through secondary damage.
- The immune response to TBI can repair or exacerbate damage, and dysregulation can cause chronic tissue damage and neuroinflammation.

**Risk Factors affecting elevated immunological sequel after traumatic injury**

- Long-term TBI risk factors: age, education, traumatic axonal injury, depression, head impact direction, inflammatory responses, and molecular factors.

**Immunopathogenesis**

- Chronic inflammation can cause neuronal death and neurological impairment in TBI. Exaggerated immune responses can result in neuroinflammation and degeneration.
- Activated microglial cell and infiltrated macrophage initiate neuroinflammatory in TBI, causing neuronal death. Blood–brain barrier changes increase inflammation.

**Current management**

- TBI requires immediate and precise management due to its complex manifestations. Supportive care is the only available treatment option, with a focus on preventing secondary consequences.
- Neuropsychiatric complications are common and may require professional help. Therapeutic interventions and products attain neuroprotection and promote brain remodeling, improving outcomes and reducing costs.

**Preventive measures**

- Preventive measures such as protective gear in vehicles and helmets, as well as increased awareness of TBI and concussion management, can reduce the number of TBI victims.

**Future directions & perspective**

- Immunotherapy and personalized approaches hold promise for TBI treatment, including modulating chemokine signaling and phenotypic screening. Clinical trials and biomarker-based diagnostics are crucial.
